# Medication Therapy Problems in Hospitalized Patients with Decreased Kidney Function Across the Spectrum of Kidney Disease: A Scoping Review

**DOI:** 10.3390/jcm15072606

**Published:** 2026-03-29

**Authors:** Tomona Iso, Danielle Antwan, Melanie Galima, Monarc Manlongat, Safer Marogy, Redi Ndrekaj, Lisa Hong

**Affiliations:** School of Pharmacy, Loma Linda University, 24745 Stewart Street, Loma Linda, CA 92350, USA

**Keywords:** medication therapy problems, hospitalized patients, kidney diseases, PQA classification framework, PCNE classification framework

## Abstract

**Background**: This scoping review aimed to identify gaps in the literature regarding medication therapy problems (MTPs) among hospitalized adults with decreased kidney function. Specifically, it aimed to answer the following questions: (1) What types of MTPs have been reported? (2) What is the reported prevalence of MTPs? (3) Do MTPs differ by type of kidney disease? (4) What gaps exist regarding MTPs and pharmacists’ involvement? **Methods**: Studies involving adult patients with decreased kidney function that investigated MTPs were included. Studies focused exclusively on post-transplant care, chemotherapy, or a single MTP type were excluded. Literature searches were conducted in PubMed, EMBASE, Cochrane Library, Web of Science, and International Pharmaceutical Abstracts. Two independent reviewers screened and extracted data, with a third reviewer resolving discrepancies. All identified MTPs were re-categorized using the Pharmacy Quality Alliance (PQA) framework and the Pharmaceutical Care Network Europe (PCNE) classification. **Results**: A total of 23 studies met the inclusion criteria, including two conference proceedings, encompassing 7151 patients. The most common MTP framework was the PCNE classification (13 studies, 57%). Reclassification using the PQA yielded 10,596 MTPs, most frequently “Safety—dosage too high” (*n* = 2464) and “Effectiveness—dosage too low” (*n* = 2262). Reclassification using the PCNE yielded 11,574 MTPs, most frequently “Drug selection” (*n* = 6974) and “Dose selection” (*n* = 2636). All studies involved patients with chronic kidney disease (CKD), and two also included acute kidney injury (AKI). **Conclusions**: Dosage-related MTPs were most prevalent among hospitalized patients with decreased kidney function. Variability in MTP definitions, limited representation of patients with AKI and AKD, and minimal reporting on pharmacists’ roles reveal important gaps. Addressing these gaps through standardized MTP classification and further research in understudied kidney disease populations may enhance patient safety and support clinical pharmacists’ contributions to optimizing medication safety across the kidney disease continuum.

## 1. Background

The concept of medication therapy problems (MTPs), originally introduced as “drug-related problems” in the 1990s, is defined as an undesirable patient experience involving drug therapy that actually or potentially interferes with a desired patient outcome (Strand 1990, Johnson 1995) [1,2,3]. While different terminologies, such as drug-related problems, drug therapy problems, medication-related problems, and medication therapy problems, have been used, the Pharmacy Quality Alliance (PQA) and the American College of Clinical Pharmacy (ACCP) recommend the term MTPs [4,5]. Medication therapy problems are often reported as part of comprehensive medication management, which aims to optimize medication use and improve patient health outcomes using a patient-centered approach and individualized care plans [5,6]. Previous research reported that pharmacists identified a mean of 4 ± 2 MTPs per patient through comprehensive medication management services [7]. The economic burden of MTPs is also huge on patients and healthcare systems. In 1995, Johnson and Bootman reported that more than 40% of patients who received medication would have MTPs, with an annual cost of drug-related morbidity and mortality in the ambulatory setting of $77 billion [2]. This estimate was updated by Watanabe et al. in 2016 to $528 billion annually [8].

Decreased kidney function occurs along a continuum and can be classified as acute kidney injury (AKI), acute kidney disease (AKD), or chronic kidney disease (CKD), since the development of AKI can ultimately result in CKD and kidney failure [9,10]. Medication management in patients with decreased kidney function requires careful consideration because they often take multiple medications, need to avoid nephrotoxic medications, and require kidney dose adjustments. One study reported that 33.5% of patients receiving kidney replacement therapy (KRT) had pharmacist-identified medication dosing problems, and another found increased odds of experiencing MTPs among patients with kidney failure during ICU stays [11]. Although patients with decreased kidney function have a higher risk of having MTPs, most studies have been conducted in ambulatory settings, such as dialysis centers, and evidence of MTPs specific to patients across the full spectrum of decreased kidney function is limited. Furthermore, previous studies used various MTP classification systems, such as Strand et al.’s original classification, the Pharmacy Quality Alliance (PQA) Medication Therapy Problem Categories Framework, the Pharmaceutical Care Network Europe (PCNE) Classification for Drug-Related Problems, or a self-developed classification, making it difficult to comprehensively evaluate MTPs among patients with decreased kidney function [1,4,12,13].

The objective of this scoping review was to identify gaps in the existing literature regarding MTPs among hospitalized adult patients with decreased kidney function. Specifically, this review aimed to answer the following questions. Among adult patients with decreased kidney function: (1) What types of MTPs have been reported? (2) What is the reported prevalence of MTPs? (3) Do MTPs differ by type of kidney disease? (4) What gaps exist in the literature regarding MTPs and pharmacists’ involvement?

## 2. Methods

This scoping review was conducted in accordance with JBI methodology for scoping reviews and the Preferred Reporting Items for Systematic Reviews and Meta-Analyses for Scoping Reviews (PRISMA-ScR), and its title was registered with Open Science Framework (https://doi.org/10.17605/OSF.IO/DFBGN) (accessed on 10 February 2026) [14,15].

### 2.1. Eligibility Criteria

This scoping review included studies involving adult patients ≥ 18 years with decreased kidney function due to AKI, AKD, or CKD, where MTPs were identified or reported. Studies published in English from 1990 to January 2024 were included, as this was when the terminology was first introduced, and MTPs were recognized as a universal problem among patients receiving medications. All study designs and gray literature, such as abstracts, conference proceedings, dissertations, government documents, and policy documents, were considered for inclusion. Studies were not limited by the MTP classification system, content, or duration of follow-up, nor by patient gender, race, or country. Only studies conducted in inpatient settings were included; studies conducted solely in outpatient settings, such as physicians’ offices, dialysis centers, home health, nursing homes, and community pharmacies, were excluded. Studies focused exclusively on post-transplant or chemotherapy management, and those reporting only one type of MTP were also excluded. Systematic reviews and meta-analyses meeting eligibility criteria were excluded but used to identify additional studies.

### 2.2. Search Strategy

The search strategy aimed to identify both published and unpublished studies using a three-step approach. First, an initial search of PubMed was undertaken to identify relevant articles. The text words in the titles and abstracts, along with the index terms used to describe the articles, were used to develop a full PubMed search strategy (Supplementary S1). This strategy, including all identified keywords and indexed terms, was adapted for each database and information source. The reference lists of all included sources were screened for additional studies. The following databases were searched: PubMed, EMBASE, Cochrane Library, Web of Science, and International Pharmaceutical Abstracts.

All identified citations were imported into EndNote 21 (Clarivate Analytics, Philadelphia, PA, USA), and duplicates were removed. Titles and abstracts were screened for eligibility independently by two reviewers. Potentially relevant sources were retrieved in full and imported into the JBI System for the Unified Management, Assessment and Review of Information (JBI SUMARI; JBI, Adelaide, Australia). The full text of selected citations was then assessed in detail against the eligibility criteria by two independent reviewers. Reasons for exclusion were recorded. Any disagreements at each stage of the selection process were resolved by a third independent reviewer.

### 2.3. Data Extraction and Analysis

The data, including article citation information, patient demographics, MTPs, medications, and interventions, were extracted by two independent reviewers using Research Electronic Data Capture (REDCap; Vanderbilt University, Nashville, TN, USA) hosted at Loma Linda University [16,17]. Any disagreements between the two independent reviewers were evaluated by a third reviewer, who reviewed the original studies and consulted with the initial reviewers to clarify discrepancies. Disagreements that could not be resolved by the third reviewer were subsequently discussed among the investigators until consensus was reached. All identified MTPs were recategorized using the PQA framework and the PCNE classification version 9.1 at the “Causes” primary domain [4,13]. The PQA framework was applied to standardize the description of MTP categories across studies, which classifies MTPs into four primary domains: indication, effectiveness, safety, and adherence, with specific subcategories within each domain. This framework is commonly used in the context of comprehensive medication management and quality measurement initiatives. The PCNE classification was additionally applied because it was the most commonly used framework among the included articles; this decision was made post hoc. The PCNE classification is a structured system widely used in international research and clinical practice to categorize MTPs, their causes, interventions, and outcomes. The PCNE framework includes multiple hierarchical domains, allowing for detailed documentation of MTPs and the underlying causes. When a study reported combined MTP categories, the MTPs were divided proportionally between the relevant categories. For example, if a study reported 10 MTPs under “inappropriate dose” that included both “dose too low” and “dose too high” according to the PQA categories, these were recategorized as 5 MTPs in “dose too low” and 5 MTPs in “dose too high”. For recategorization using the PQA framework, medication errors such as transcription errors and MTPs without specific reasons (e.g., “other” category) were excluded, as the PQA framework does not provide a category for these problems, in contrast to the PCNE classification. A narrative summary accompanied the tabulated results. All data were managed and summarized using R (version 4.5.1; R Foundation, Vienna, Austria) and RStudio (version 4.5.1 +513; Posit Software, PBC, Boston, MA, USA).

## 3. Results

**Figure 1:**
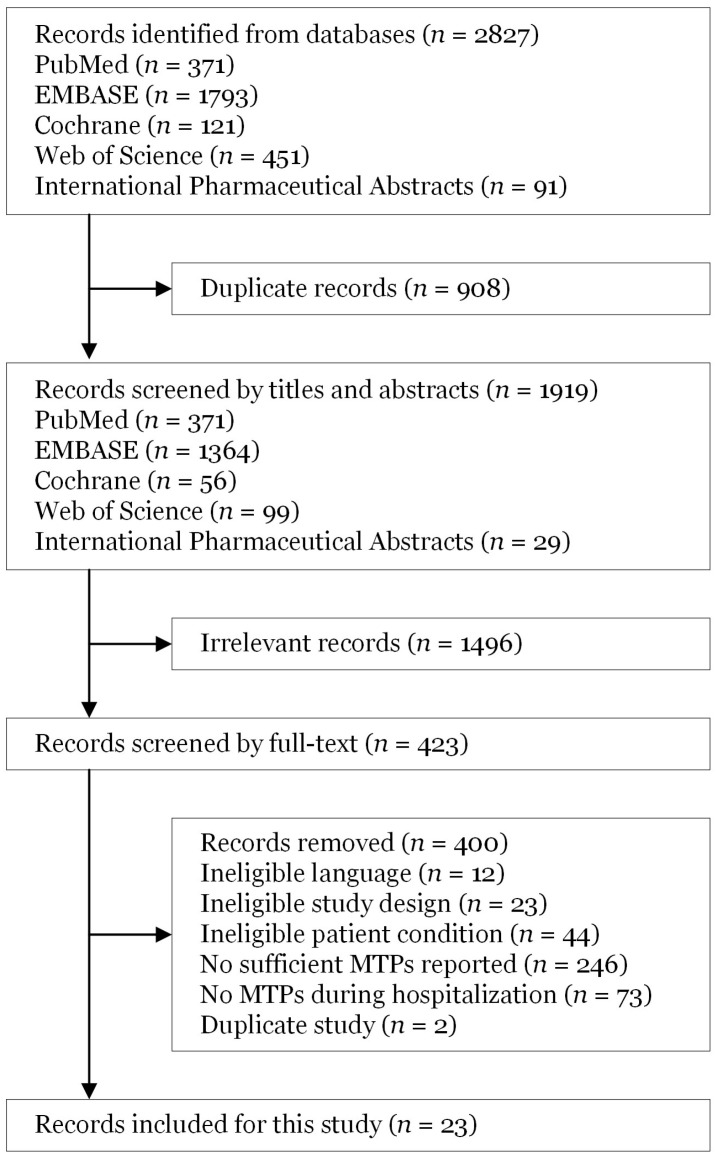
Screening flowchart diagram. MTPs, medication therapy problems.

A total of 2827 articles were identified, and after removing duplicates, 1919 articles were screened by title and abstract (Figure 1). Of those, 423 were further screened by their full text. After full-text screening, 23 articles were deemed eligible, including 2 conference proceedings [18,19,20,21,22,23,24,25,26,27,28,29,30,31,32,33,34,35,36,37,38,39,40]. Table 1 summarizes the characteristics of the included studies, including study design, types of kidney disease, patient populations, and the classification frameworks used to report medication therapy problems. Among the included studies, 17 (74%) were prospective observational studies, 4 (17%) were retrospective observational studies, and 2 (9%) were randomized controlled trials. Collectively, 7151 patients were represented across 14 countries. The most commonly used MTP framework was the PCNE classification (*n* = 13, 57%), and none used the PQA framework.

### 3.1. Types and Prevalence of MTPs Reported

The types and prevalence of MTPs are summarized in Table 2 and Table 3, with a wide range of MTPs documented across all included studies.

#### 3.1.1. MTPs Using PQA Framework

Using the PQA framework, a total of 10,596 MTPs (1.5 per patient) were reported across 23 studies (Table 2). The most common MTP category was “Safety—dosage too high,” with 2464 MTPs reported across all 23 studies. The second most common category was “Effectiveness—dosage too low,” with 2262 MTPs reported in 22 studies. The least common category was “Adherence—cost,” with only 56 MTPs reported in 6 studies.

#### 3.1.2. MTPs Using PCNE Classification

Using the PCNE classification, a total of 11,574 MTPs (1.6 per patient) were reported across 23 studies (Table 3). The total number of MTPs differed between the PQA and PCNE classification frameworks because the PCNE classification includes medication error-related MTPs and an “other” category. The most common MTP category was “Drug selection,” with 6974 MTPs reported in all 23 studies. The next most common category was “dose selection”, with 2636 MTPs reported across all 23 studies.

Most studies reported multiple MTPs per patient, with dose-related safety and effectiveness issues appearing in nearly all studies. Together, these findings show that dose-related and drug selection problems dominate the MTP landscape among adults with decreased kidney function.

### 3.2. MTPs by Type of Kidney Disease

Across the included studies, the majority had CKD, totaling 5676 patients (79%) from 22 studies. Only 216 (3%) from 4 studies were undergoing kidney replacement therapy, 196 (3%) from 2 studies had AKI, and another 1063 (15%) had an unspecified type of kidney impairment. No studies specifically reported AKD. No comparisons were made across kidney disease types.

### 3.3. Gaps Regarding MTPs and Pharmacists’ Involvement

The scoping review identified several notable gaps, including a lack of standardization in MTP definitions, with none using the PQA framework, and limited representation of AKD (0 studies), AKI (2 studies), and KRT (4 studies) populations. Among the 23 included studies, 14 (61%) reported interventions by healthcare professionals, including pharmacists, addressing identified MTPs, accounting for a total of 4361 interventions. Of these, 3296 interventions (76%; reported in 14 studies) were implemented, 638 (15%; 9 studies) were not implemented, 14 (0.3%; 2 studies) involved no intervention, and 413 (9%; 5 studies) had unknown resolution. Only 3 studies evaluated the association between pharmacist interventions and clinical outcomes, including 90-day rehospitalization (*n* = 1), 90-day emergency department or urgent care visits (*n* = 2), and quality of life (*n* = 1). These studies did not find statistically significant differences in rehospitalization or emergency/urgent care visits; however, one study reported a significant improvement in quality of life.

## 4. Discussion

### 4.1. Types of MTPs Reported in the Literature

This comprehensive scoping review identified substantial variability in MTP classification across the literature and mapped the reported frequency of MTPs among hospitalized patients with decreased kidney function using the PQA and PCNE frameworks to describe MTPs in a standardized manner. As a scoping review, this study aimed to map and describe the existing literature rather than to quantitatively synthesize outcomes or assess causal relationships. Dosage-related problems emerged as the most consistently reported MTP category affecting both medication safety and effectiveness. Given that patients with decreased kidney function often require dose reductions, we anticipated that “dose too high” would be more prevalent than “dose too low.” However, the results showed that both categories contributed almost equally to the total number of dosage-related MTPs. This finding may reflect challenges in optimizing medication dosing in this patient population and highlights potential opportunities for pharmacist involvement, such as initial dose selection; dose adjustment in the setting of rapidly changing kidney function; monitoring for signs or symptoms of under- or overdosing; and therapeutic drug monitoring. It should be noted that some studies reported dosage-related problems as a combined category (“dose too low or too high”), and in such cases, the counts were proportionally divided between the two categories. Using the PCNE classification, the most common MTPs were related to drug selection. This discrepancy likely reflects differences in how the two frameworks categorize MTPs. In the PQA framework, adverse medication events are assigned to a separate category, whereas in the PCNE classification, they are not explicitly categorized and may instead be classified under drug selection problems. The predominance of dosing and drug selection problems represents areas where clinical pharmacists may contribute through medication reconciliation, dose adjustment for individual kidney function, prospective medication review, and interdisciplinary collaboration during hospitalization.

### 4.2. Prevalence of MTPs

Previous studies in outpatient settings involving patients with CKD and those receiving KRT have reported a wide range in MTP prevalence, from 1.4 to 5.4 per patient [7,41,42,43]. In contrast, the inpatient-focused studies in this review demonstrated a lower prevalence of MTPs at 1.5 per patient using the PQA framework and 1.6 per patient using the PCNE classification. Differences between outpatient and inpatient settings may reflect variations in how medication reviews are conducted and the extent to which clinical pharmacy services are integrated into routine care. The slight difference between the framework estimates is likely due to the inclusion of medication errors and an “other” category in the PCNE classification. These findings indicate that reported MTP prevalence varies depending on the classification framework used, reinforcing the importance of using consistent frameworks when interpreting reported MTP frequencies across studies. Given the variability in study design, MTP detection methods, and classification frameworks, these averages should be interpreted as descriptive approximations rather than pooled prevalence estimates. Regardless of the framework used, the high prevalence of dose-related MTPs suggests clear opportunities for pharmacists to contribute meaningfully.

### 4.3. Differences in MTPs by Type of Kidney Disease

Similar to outpatient studies, the included studies focused predominantly on patients with CKD (79%). Only a small number of studies reported MTPs during the acute phase of kidney dysfunction, limiting the ability to draw robust conclusions regarding differences in MTP types or prevalence across specific kidney disease categories. Additionally, some studies did not specify the type of decreased kidney function or explicitly report KRT use, resulting in 15% of patients being categorized as having an unknown type of decreased kidney function. This limitation suggests that the actual number of patients with AKI and KRT may be higher than reported. Nevertheless, descriptions of MTPs among patients with AKI or AKD remain scarce. Given that AKI can progress to CKD and kidney failure, inpatient medication management represents a critical opportunity to prevent long-term complications. Clinical pharmacists may play an essential role in this transitional phase by identifying and addressing MTPs early during hospitalization. Future studies should address this gap by more clearly characterizing MTPs in acute settings where kidney function is unstable, and patients are highly susceptible to dosing errors and medication-related harm.

### 4.4. Gaps in the Literature Regarding MTPs and Pharmacists’ Involvement

This review identified several important gaps in the literature. There is substantial heterogeneity in MTP definitions and classification frameworks, which complicates comparisons across studies and limits synthesis. Standardized reporting of pharmacist-identified MTPs and interventions would enhance the ability to evaluate the impact of clinical pharmacy services. Also, patients with AKI and AKD remain underrepresented in the literature, despite being at high risk for medication-related harm. Finally, although several included studies described pharmacist-led identification and management of MTPs, reporting of intervention processes and outcomes was inconsistent. Although pharmacist interventions were frequently implemented when reported, only a small number of studies evaluated their association with clinical outcomes. This limited reporting makes it difficult to determine the extent to which pharmacist interventions translate into measurable clinical benefits. Future studies should prioritize evaluating pharmacist-led interventions in these populations and assessing downstream clinical outcomes such as adverse drug events, length of stay, and progression of kidney disease.

### 4.5. Strengths and Limitations

One major strength of this review was the inclusivity of the search strategy. We searched 5 databases and included both published and gray literature, which enabled us to capture a broad spectrum of studies and minimize publication bias. This approach also provided a more representative picture of MTPs among patients with decreased kidney function and reflected diverse geographical locations and inpatient settings. Furthermore, the use of both the PQA framework and PCNE classification to recategorize all identified MTPs standardized reporting and improved comparability across studies. This allowed for a more nuanced understanding of how different studies conceptualized and interpreted MTPs, resulting in a comprehensive synthesis of the data. Another strength was the rigorous review process, in which two independent reviewers assessed each article and extracted data, with a third reviewer resolving any discrepancies.

Despite these strengths, several limitations must be acknowledged. First, the lack of universal MTP definitions and standardized reporting measures across studies hindered objective data collection and made the recategorization process more dependent on reviewer interpretation. Second, 2 of the 23 included studies were available only as conference proceedings. While the inclusion of gray literature helps minimize publication bias, the limited level of detail in these sources may have affected the reliability of the extracted data. Third, some studies did not provide detailed breakdowns within a category, for example, reporting dosing problems without distinguishing between “dose too low” and “dose too high.” In such cases, this review proportionally divided the reported counts between the applicable categories as a pragmatic approach for recategorization. However, this proportional allocation represents an assumption rather than an empirically derived distribution, and the actual proportions within the original studies may differ. Therefore, the relative frequencies of categories such as “dose too low” and “dose too high” might lead to under- or overestimation and should be interpreted cautiously. Finally, this review included only studies published in English, which may have resulted in the exclusion of relevant studies published in other languages.

This scoping review mapped the reported MTPs among hospitalized patients with decreased kidney function, and the findings of this review may help clinicians recognize common patterns of MTPs in this population. Dosage-related problems affecting both medication safety and effectiveness were common across studies, reflecting the complexity of medication management in this population. Another commonly reported MTP was medication selection, particularly the use of medications with nephrotoxic potential. This highlights areas where careful medication review and interdisciplinary collaboration, including the involvement of clinical pharmacists, may help identify and address MTPs in hospitalized patients with kidney dysfunction. However, variability in MTP definitions and classification frameworks, limited detail in some studies, underrepresentation of patients with AKI and AKD, and minimal reporting of pharmacist involvement or patient-level outcomes revealed important gaps in the literature. These findings highlight critical opportunities for clinical pharmacists to optimize medication safety and effectiveness across the continuum of kidney disease. Given the heterogeneity in study designs, MTP definitions, and reporting methods across the included studies, the findings should be interpreted as a descriptive overview of reported MTPs rather than precise estimates of their overall prevalence. Future research should prioritize improving consistency in MTP classification and exploring MTPs in understudied populations, as well as prospective evaluation of pharmacist-led interventions in patient-centered outcomes to enhance patient safety and optimize clinical outcomes.

## Figures and Tables

**Table 1:** Characteristics of included articles and MTP classification frameworks.

Author Year	Study Design	Country	Type of Kidney Disease	Number of Patients	Age, Year (Mean ± SD)	Male, *n* (%)	Framework
Blix 2006 [20]	Prospective Observational Study	Norway	CKD	201	80 ± 10	686 (85%)	PCNE
AbuRuz 2013 [18]	Prospective Observational Study	Jordan	CKD	130	56 ± 18	74 (57%)	AbuRuz et al.’ classification
Holm 2015 [25]	Prospective Observational Study	Norway	CKD	79	79 ± 10	44 (56%)	Ruths et al.’s classification
Ramadaniati 2016 [31]	Prospective Observational Study	Indonesia	CKD	105	49 *	48 (46%)	PCNE
Adibe 2017 [19]	Prospective Observational Study	Nigeria	CKD	287	72 ± 8	116 (40%)	PCNE
Tuttle 2018 [38]	RCT	USA	CKD	141	69 ± 11	74 (52%)	Medication Discrepancy Tool
Dvorackova 2019 [22]	Prospective Observational Study	Czech Republic	CKD	1850	54 *	1169 (63%)	PCNE
Garedow 2019 [23]	Prospective Observational Study	Ethiopia	CKD	103	46 ± 18	72 (70%)	Cipolle et al.’s classification
Tesfaye 2019 [37]	Retrospective Observational Study	Australia	CKD	204	83	125 (61%)	N/A
Roy 2020 [32]	Prospective Observational Study	India	CKD	200	58 ± 20	132 (66%)	PCNE
Savitha 2020 [33]	Prospective Observational Study	India	CKD	833	54 ± 13	468 (56%)	PCNE
Subeesh 2020 [35]	Prospective Observational Study	India	CKD	160	51 ± 15	114 (71%)	PCNE
Liu 2021 [26]	Retrospective Observational Study	China	CKD	87	49 ± 15	47 (54%)	PCNE
Song 2021 [34]	RCT	South Korea	CKD	100	54 ± 17	60 (60%)	PCNE
Peri 2022 [29]	Prospective Observational Study	Indonesia	CKD	83	49 ± 12	54 (65%)	PCNE
Pehlivanli 2023 [28]	Prospective Observational Study	Turkey	CKD	269	59 ± 16	139 (52%)	PCNE
Zhang 2023 [40]	Prospective Observational Study	China	CKD	914	60 ± 18	350 (38%)	PCNE
Usman 2023 [39]	Retrospective Observational Study	Nigeria	AKI, CKD	688	53 ± 18	245 (36%)	N/A
Gharekhani 2014 [24]	Prospective Observational Study	Iran	CKD, KRT	406	51 ± 18	277 (68%)	PCNE
Possidente 1999 [30]	Retrospective Observational Study	USA	KRT	37	66 ± 13	19 (51%)	N/A
Ong 2006 [27]	Prospective Observational Study	Canada	KRT	47	68 ± 12	31 (66%)	Hepler and Strands
Dahse 2015 [21] *	Prospective Observational Study	Germany	N/A	197	81	68 (35%)	N/A
Tecen 2018 [36] *	Prospective Observational Study	Turkey	AKI, CKD, KRT	30	N/A	N/A	N/A

Studies are presented first by publication type (fully published articles followed by conference proceedings), then by type of kidney disease studied, and within these groups in chronological order by publication year. AKI, acute kidney injury; CKD, chronic kidney disease; KRT, kidney replacement therapy; MTP, medication therapy problem; N/A, not available; PCNE, Pharmaceutical Care Network Europe Classification for Drug-Related Problems; RCT, randomized controlled trial. * Data from conference proceedings.

**Table 2:** Summary of medication therapy problems recategorized by the PQA framework.

Medication-Related Needs	Medication Therapy Problem Category	Number of MTPs, *n* (%) (*N* = 10,596)	Number of Studies, *n* (%) (*N* = 23)
Indication	Unnecessary medication therapy	790 (7%)	19 (83%)
	Needs additional medication therapy	1587 (15%)	18 (78%)
Effectiveness	Ineffective medication	477 (5%)	17 (74%)
	Dosage too low	2252 (21%)	22 (96%)
	Needs additional monitoring	225 (2%)	9 (39%)
Safety	Adverse medication event	2052 (19%)	23 (100%)
	Dosage too high	2464 (23%)	23 (100%)
	Needs additional monitoring	225 (2%)	9 (39%)
Adherence	Adherence	470 (4%)	14 (61%)
	Cost	56 (1%)	6 (26%)

MTP, medication therapy problem; PQA, Pharmacy Quality Alliance. When a study reported combined MTP categories, the MTPs were divided proportionally between the relevant categories. For example, if a study reported 10 MTPs under “inappropriate dose” that included both “dose too low” and “dose too high” according to the PQA categories, these were recategorized as 5 MTPs in “dose too low” and 5 MTPs in “dose too high”.

**Table 3:** Summary of medication therapy problems recategorized by the PCNE classification.

Medication Therapy Problem Causes	Number of MTPs, *n* (%) (*N* = 11,574)	Number of Studies, *n* (%) (*N* = 23)
C1. Drug selection	6971 (60%)	23 (100%)
C2. Drug form	44 (1%)	5 (22%)
C3. Dose selection	2636 (23%)	23 (100%)
C4. Treatment duration	79 (1%)	5 (22%)
C5. Dispensing	253 (2%)	7 (30%)
C6. Drug use process	290 (3%)	8 (35%)
C7. Patient related	443 (4%)	15 (65%)
C8. Patient transfer related	423 (4%)	5 (22%)
C9. Other	435 (4%)	11 (48%)

MTP, medication therapy problem; PCNE, Pharmaceutical Care Network Europe.

## Data Availability

The data that support the findings of this study are available from the corresponding author upon reasonable request.

## Supplementary Materials

The following supporting information can be downloaded at: https://www.mdpi.com/article/10.3390/jcm15072606/s1, Supplementary S1. Search strategy.
