# Supplementary material for: Medication Therapy Problems in Hospitalized Patients with Decreased Kidney Function Across the Spectrum of Kidney Disease: A Scoping Review
Source: J Clin Med. 2026 Mar 29;15(7):2606. doi: 10.3390/jcm15072606 (PMC13073041; doi:10.3390/jcm15072606)
Supplement: Supplementary file 1 [file jcm-15-02606-s001.zip › jcm-4175948-supplementary.pdf]

Supplementary S1. Search strategy

MEDLINE (PubMed)

Search conducted: January 10, 2024

Search	Query	Record retrieved
#1	(Acute Kidney Injury[Mesh] OR "acute kidney injury"[tiab] OR AKI[tiab] OR “acute kidney disease*”[tiab] OR AKD[tiab] OR Kidney failure, Chronic[Mesh] OR “chronic kidney disease*”[tiab] OR renal insufficiency, chronic[Mesh] OR CKD[tiab] OR “end-stage renal disease*”[tiab] OR ESRD[tiab] OR kidney diseases[Mesh] OR “kidney disease*”[tiab] OR “kidney function”[tiab] OR “renal insufficiency”[tiab] OR “renal dysfunction”[tiab] OR “renal disease*”[tiab] OR Renal Replacement Therapy[Mesh] OR Renal Dialysis[Mesh] or “renal replacement therapy”[tiab] OR “dialysis”[tiab] OR “hemodialysis”[tiab])	848,927
#2	(Medication Therapy Management[Mesh] OR “medication therapy management” [tiab] OR “medication management”[tiab] OR Drug Therapy Management[Mesh] OR “drug therapy management”[tiab] OR “medication intervention*”[tiab] OR “medication appropriateness”[tiab])	8,383
#3	(MTP[tiab] OR MTPs[tiab] OR “medication therapy problem*”[tiab] OR “medication-therapy problem*”[tiab] OR “medication problem*”[tiab] OR MRP[tiab] OR MRPs[tiab] OR “medication related problem*”[tiab] OR “medication-related problem*”[tiab] OR DTP[tiab] OR DTPs[tiab] OR “drug therapy problem*”[tiab] “drug-therapy problem*”[tiab] OR DRP[tiab] OR DRPs[tiab] OR “drug related problem*”[tiab] OR “drug-related problem*”[tiab])	4,004
#4	((Adolescent[Mesh] OR Child[Mesh] OR Infant[Mesh] OR adolescen*[tiab] OR child*[tiab] OR schoolchild*[tiab] OR infant*[tiab] OR girl*[tiab] OR boy[tiab] OR boys[tiab] OR teen[tiab] OR teens[tiab] OR teenager*[tiab] OR youth*[tiab] OR pediatr*[tiab] OR paediatr*[tiab] OR puber*[tiab]) NOT (Adult[Mesh] OR adult*[tiab] OR man[tiab] OR men[tiab] OR woman[tiab] OR women[tiab]))	2,447,983
#5	#2 OR #3	12,084
#6	#1 AND #5	387
#7	#6 NOT #4	373
#8	#7 AND (1990:2023[pdat])	371
